# Personal Construction of Cough Medicine among Young Substance Abusers in Hong Kong

**DOI:** 10.1100/2012/754362

**Published:** 2012-04-19

**Authors:** Daniel T. L. Shek

**Affiliations:** ^1^Department of Applied Social Sciences, The Hong Kong Polytechnic University, Hong Kong; ^2^Public Policy Research Institute, The Hong Kong Polytechnic University, Hong Kong; ^3^Department of Social Work, East China Normal University, Shanghai 200241, China; ^4^Kiang Wu Nursing College of Macau, Macau, China; ^5^Division of Adolescent Medicine, Department of Pediatrics, Kentucky Children's Hospital, University of Kentucky College of Medicine, Lexington, KY 40506, USA

## Abstract

Although cough medicine abuse is a growing problem in many places, there is no study examining the views of young substance abusers toward cough medicine. The objective of this study was to examine personal constructions of cough medicine abusers via the repertory grid tests (*N* = 11). Several observations are highlighted from the study. First, personal constructions of cough medicine were mixed, including the benefits and harmful effects of its abuse. Second, although the informants perceived cough medicine to be addictive and harmful, they perceived cough medicine to be less addictive and less harmful than did heroin. Third, while the informants construed cough medicine to be similar to ketamine and marijuana, they also perceived cough medicine to possess some characteristics of heroin. Fourth, relative to the construed similarity between heroin and the gateway drugs (cigarette, beer, and liquor), the informants construed cough medicine to be more similar to the gateway drugs. Finally, a higher level of perceived dissimilarity between cough medicine and gateway drugs was related to a higher level of perceived harm of cough medicine abuse.

## 1. Introduction

Adolescent substance abuse is a rising problem in the global contexts. However, while much attention has been spent on the use of cannabis and amphetamine-related drugs in the past few years, less attention has been directed to the abuse of licit drugs that can be bought over the counter (i.e., OTC drugs), such as cough medicine. There are reports on the possible abuse of cough medicine in the United States and Denmark [1, 2]. According to the press release of the Students for Sensible Drug Policy [3], officials in the United States found that abuse of cough medicine containing dextromethorphan had been intensified among the youth. The findings reported by the Child and Adolescent Workgroup of the National Institute on Drug Abuse in the United States also indicated that the use of cough medicine increased in grade 10 and grade 11 students [4]. Similar warning was given by the Teen OTC and Prescription Drug Abuse [5].

It was reported that the sale for cough medicine containing opiate constituents ranked highest for nonprescription sales in the United Kingdom [6]. According to the BBC Online Network [7], some addicts in the United Kingdom consumed Nurofen Plus, which was made up of ibuprofen, and codeine, to get high. Regarding the factors contributing to the abuse of cough medicine, Darboe [8] proposed a theoretical model in which four factors were identified: availability factor, approval factor, ignorance factor, and fear factor. Based on a survey of school personnel, Darboe et al. [9] asserted that “abuse of cough syrup (Robitussin or other brands) has increased over the years and is increasingly perceived as a problem by the community” (p.633).

Cough medicine abuse is also a growing problem in Asia. The prevalence of cough medicine abuse was serious in some Asian countries [10] and codeine was illegally used for abuse purpose in Bangladesh, Malaysia, and Myanmar [11]. In Malaysia and Myanmar, cough medicine containing codeine was often used in combination with other drugs [10]. Besides the illicit use of cannabis and alcohol in Nepal, licit codeine-based medicines had continued to be abused [12]. According to a report of Medical Tribune Online [13], the situation in Malaysia was so bad that the Drug Control Authority banned all codeine-based cough preparations starting January 1, 2003. Besides, increased abuse of cough mixture was reported in Japan [14, 15] and India [16, 17]. Mattoo et al. [18] studied the sociodemographic and clinical profiles of 46 patients abusing codeine-containing cough syrups (CCS). Findings showed that friends and curiosity were the main reasons for abuse, and “the combination of an opioid and a sympathomimetic agent in the CCS may cause a special, distinct euphoric effect” (p.1783).

With specific reference to Hong Kong, several sources of information can give an indication of the severity of the problem. The first source of information is based on the Central Registry of Drug Abuse [19]. Generally speaking, abuse of cough medicine has been on the rising trend in past few years, although there are some fluctuations. Another source of information is from the school surveys regularly conducted by the Narcotics Division [20–24]. In a recent school survey, it was found that cough medicine abuse was ranked fourth in the drugs commonly abused by psychotropic drugs-taking students (following ketamine, cannabis, and ecstasy) with 26.4% of the students abusing the drug [25].

The third source of information is based on the studies conducted or sponsored by the Narcotics Division of the Security Bureau of the Hong Kong Government. For example, in a study of young drug abusers, Narcotics Division [26] showed that heroin, cannabis, and cough medicine were the three most commonly abused drugs. Narcotics Division [27] reported that among the 1,110 respondents responding to the question of “Do you know what is the most commonly abused drug in Hong Kong?”, 16.8% of the respondents perceived cough medicine to be a drug commonly abused. With reference to “other drugs” perceived by the respondents to be commonly abused in Hong Kong, 48.5% of the respondents perceived cough medicine to be a drug commonly abused by people in Hong Kong. In another study of psychoactive substance abusers of PS33, Lai [28] showed that cough medicine was a major primary drug of abuse among detoxified and nondetoxified subjects. With reference to students' use of a drug within 30 days and knowledge of other drug users, Day and Lee [29] showed that cough medicine was a drug commonly abused by students.

The fourth source of information is based on research conducted by academics and psychiatrists. Based on a retrospective study of 27 patients, Lam et al. [30] showed that the major psychiatric presentations of cough medicine abuse included acute organic brain damage, schizophreniform psychosis, and affective episode, and remarked “cough mixture misuse has become a focus of concern in Hong Kong since the 1980s” (p.1375). In a study comparing high school students with incarcerated offenders in Hong Kong, Wong et al. [31] found that cough medicine abuse was related to attitudes and intentions to try and peer drug use. The final source of information comes from the research conducted by the nongovernmental organizations (NGOs). With the intensification of the problem of adolescent substance abuse, many NGOs have conducted researches on adolescent substance abuse that have covered cough medicine abuse. For example, Caritas Aberdeen Outreaching Team [32] showed that among those who had been to rave parties and discos (*N* = 151), 12 respondents (7.9%) had abused cough medicine.

One observation that can be highlighted from the above studies is that studies on attitudes to and beliefs about cough medicine are almost nonexistent although there are some studies on attitudes to substance abuse in general. In a study conducted by the Narcotics Division on public attitude towards substance abuse [27], 76.4% of the respondents agreed that “once people start taking drugs it can be too late”, and 96.3% of the respondents agreed that “drugs are dangerous and easily addictive”. Another study conducted by the Narcotics Division [26] also showed that 26.2% of the respondents agreed that they could control their heroin-taking habit and not addict to the drug. Based on qualitative findings, Narcotics Division [33] also reported that psychotropic substances abusers believed that they could control the dosage of drug intake to produce the desired effect without losing control.

Students' attitudes toward substance abuse were also examined by Lau [20]. The following findings were reported: 87.4% agreed that “taking heroin is harmful to one's health”; 84.4% agreed that “taking substance like pills, cannabis, cough medicine, solvent thinner is harmful to health”; 81.9% agreed that “taking ecstasy/ketamine is harmful to health”; 84% agreed that “drug abuse destroys your future”; 17.6% agreed that “nowadays, taking pills and cannabis is a hobby, just as smoking”; 15.7% of the respondents agreed that “I can control my heroin taking habit to make heroin taking not addictive”; 19.5% of the respondents agreed that “I can control my habit of taking ecstasy/ketamine to make ecstasy/ketamine taking not addictive”; 20.7% agreed that “I can control my drug taking habit to make substance abuse not addictive.”

In another study conducted by Chiu and Wong [34], attitude and values of children and adolescents toward substance abuse were examined. The researchers examined values about substance abuse via three items: “because adolescents abusing drugs just want to pursue happiness, there is no big deal about this”; “adolescents who want to have fun around should not be fearful about drugs”; “if you do not hurt anybody, there is no harm in taking drugs.” The conclusion of the study was that while 67% of the participants held “low” deviant values, 29.1% and 4% had “medium” and “high” deviant values, respectively. However, one limitation of the study is that it is not clear how the different levels of deviant values were operationally defined.

As there is no scientific study on cough medicine abusers' attitudes toward cough medicine, the objective of this study was to examine young substance abusers' personal constructions of cough medicine in relation to different types of drugs and nondrugs via the repertory grid test based on personal construct psychology. According to Kelly [35], the universe is an ongoing process which can only be understood in terms of construction and reconstruction (e.g., from a “safe” drug to “dangerous” drug) through the personal constructs of a person which refer to the world views, interpretations, and deductions about life. Actually, personal constructs are transparent templates, through which the external reality is understood, or categories of thoughts by which an individual construes or interprets his personal world (e.g., “becoming high” versus “not becoming high” after consumption). Personal construct psychology has been used in different areas, including psychotherapy, personality assessment, organizational psychology, and education.

The repertory grid test is an assessment method closely related to personal construct psychology that assesses the personal construct system of an individual [36–38]. There are three unique features of the repertory grid test. First, the assessment method assesses the individual's subjective worldview from the perspective of the individual (i.e., using the language of the informant). Second, the assessment method can yield data that can be analyzed via quantitative as well as qualitative methods. Finally, the repertory grid test is a very flexible method (e.g., adjusting the number of elements and constructs) that can yield very rich information.

In the Chinese context, Shek and Lam [39] used the repertory grid test to examine the effectiveness of a pioneering drug prevention program in Hong Kong. Group analyses of the grid data of the 30 informants showed that the selves of the participants improved in several areas after joining the program. In sum, the informants psychologically identified themselves (i) more with the “Ideal Self”; (ii) less with “A Drug Addict”; (iii) more with “A Successful Person”; (iv) less with “An Unsuccessful Person.” Further qualitative findings showed that there are significant changes in the selves of the informants after joining the program. For example, one informant perceived herself as playful, not understanding oneself, having no future, having no goals, failure, nonpersuasive, having no self-control, having no power, and not listening to others before joining the program. However, the informant perceived herself to have the following characteristics after joining the program: mature, understanding herself, thinking about the future, having goals, successful, persuasive, having self-control, having power, and accepting others' views. An examination of the grid data showed that the informant perceived herself to be very close to a drug addict and an unsuccessful person before joining the program. However, she began to identify herself with the ideal self and a successful person after joining the program.

## 2. Methods

In the present study, a total of 11 informants were invited to complete the grid on a convenience base. All informants were single males aged between 15 and 24 years. Informed consent was obtained from all participants before the commencement of the study. The following 15 elements were used to elicit the constructs.

Element 1: heroin.
Element 2: cough medicine.
Element 3: organic solvent.
Element 4: marijuana.
Element 5: tranquilizers.
Element 6: “ice”.
Element 7: ecstasy.
Element 8: cocaine.
Element 9: depressants.
Element 10: ketamine.
Element 11: cigarette.
Element 12: beer.
Element 13: liquor.
Element 14: essence of chicken.
Element 15: chewing gum.

For each participant, 10 constructs were elicited via the triadic method. For every triad (i.e., three elements), the participant was asked to construct in which most important way two elements were alike but differed from the third one. After the constructs were elicited, the participant was then asked to rate all the elements on the construct (i.e., along the construct and contrast poles) on a 6-point scale, with 4 to 6 represented the construct pole and 1 to 3 represented the contrast pole. Besides the elicited constructs, there were two supplied constructs (addictive versus nonaddictive and lethal versus nonlethal). The raw data for each grid (15 elements by 12 constructs) were analyzed by the INGRID 72 program [40, 41].

In the output of the analysis, there is a section on the distances between pairs of elements. Distances between elements represent the psychological distances between elements, with a minimum value of 0, a mean of 1, and it seldom exceeds the value of 2. Therefore, if the distance between a pair of elements is close to zero, it means that they are seen as similar in the psychological space of the person. On the other hand, if the distance between a pair of elements is close to 2, it means that they are seen to be dissimilar in the psychological space of the informant. Based on the distances between elements, the participants' perceptions of cough medicine in relation to different types of drugs and nondrugs can be revealed. Shek and Lam [39] showed that repertory grid test is a very sensitive method to capture the subjective worldviews of the informants.

## 3. Results

Repertory grid test data can be analyzed by both quantitative and qualitative methods [42]. For quantitative data analyses, many computer programs are available [43]. For the present study, INGRID 72 was used to analyze the data, the details of which can be seen in Slater [40, 41]. INGRID 72 generates a wide range of information, including distances between elements in the psychological space of the informant. According to Norris and Makhlouf-Norris [44], distance between two elements, can be regarded as the degree of similarity or dissimilarity between two elements and this measure can be regarded as an indicator of a person's degree of identification with an element. Stanley [45] used distances between elements to assess psychological and social alienation in young offenders. Local researchers have also used distances between elements to explore the self-identity systems of drug addicts, mental patients, and adolescents after joining a positive youth development program [39, 46, 47].

### 3.1. Personal Construction of Cough Medicine by the Abusers

The informants' construction of the characteristics of cough medicine is summarized in Table 1. An examination of the findings showed that the related constructs could be categorized into four categories. The first category is on the psychological consequences of abusing cough medicine. All informants construed that cough medicine contributed to changes at the cognitive (e.g., poor memory), affective (e.g., feeling happy), and behavioral (e.g., increase in motor activities) levels. Actually, most of the attributes related to cough medicine were perceived psychological benefits of taking cough medicine. The second category was on the addictive nature of cough medicine, with several informants construed that cough medicine was addictive. The third category was on the harmful effects of cough medicine. The final category was related to other aspects of cough medicine, with some informants construed cough medicine as cheap and accessible. 

With reference to the supplied constructs, the informants perceived cough medicine to be additive (mean = 4.91, SD = 0.94) and harmful (mean = 4.73, SD = 1.01). However, analyses using Wilcoxon Test showed that the informants perceived cough medicine to be less addictive (mean = 5.82, SD = 0.60 for heroin, *z* = −2.16, *P* < .05) and less harmful (mean = 6.00, SD = 0.00 for heroin, *z* = 2.57, *P* < .02) than heroin.

Two observations can be highlighted with respect to the construed characteristics of cough medicine. First, the constructions were mixed where the informants construed both positive and negative consequences of taking cough medicine. Second, some participants perceived that cough medicine was cheap and easily accessible.

### 3.2. Cough Medicine versus Other Drugs

To what extent cough medicine is perceived to be similar to or different from other drugs? An examination of the findings in Table 2 showed that the informants perceived that cough medicine was most similar to ketamine and marijuana and least similar to organic solvent. In other words, the informants appeared to see cough medicine as a kind of “soft drugs.” Nevertheless, results also showed that the informants construed that there was no big difference between cough medicine and heroin. This finding is interesting because it is commonly believed that heroin is very different from cough medicine. In short, the informants construed that cough medicine was similar to soft drugs that also had some characteristics of heroin.

**Figure 1:**
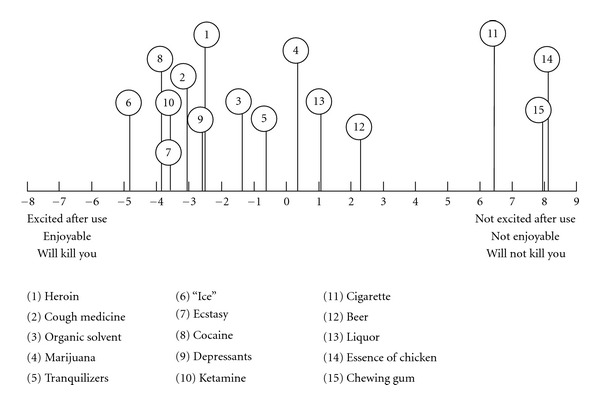
Representation of cough medicine and other drugs and non-drugs in the psychological space in terms of the first principal component (informant no. 7).

An illustration based on case no. 7 can give some ideas on the informant's perception of cough medicine and other drugs. The informant was aged 28, and he had tried heroin, marijuana, tranquilizers, ecstasy, and cough medicine. He began abusing cough medicine at the age of 14. His usual consumption of cough medicine was once every day, and he had abused cough medicine for more than once every day in the past three months. On the day of interview, he claimed that he had just consumed a bottle of cough medicine before attending the interview. With reference to Table 3, the informant construed the consumption of cough medicine generating the following characteristics: feeling comfortable (2 times), feeling “wing,” feeling “high,” feeling gratified, feeling excited, relatively more toxic, feeling less painful (2 times), and having greater harm. The informant's representation of cough medicine and other drugs and non-drugs in his psychological space in terms of the first principal components analysis is presented in Figure 1. In the figure, it is evident that the informant construed the different types of drugs to be relatively the same (that would give “high” and “gratified” feelings, but they would kill you) that were very different from the nondrugs. 

### 3.3. Cough Medicine and Other Drugs versus Nondrugs

Several nondrug elements were utilized in the present study. These included cigarette, beer, liquor, essence of chicken, and chewing gum. Because cigarette, beer, and liquor are commonly regarded as gateway drugs, group analyses were conducted to examine how cough medicine was construed to be similar or different from these gateway drugs. Several observations could be highlighted from the following findings.

**Figure 2:**
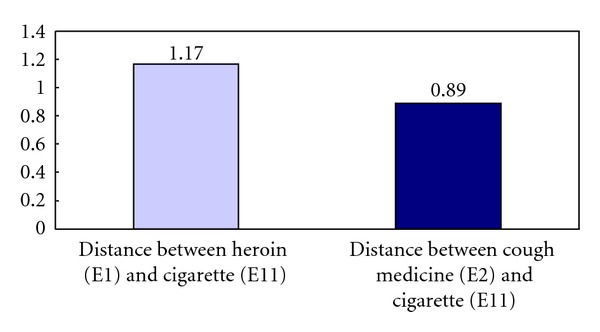
Mean distance between heroin (E1) and cigarette (E11) versus mean distance between cough medicine (E2) and cigarette (E11).

**Figure 3:**
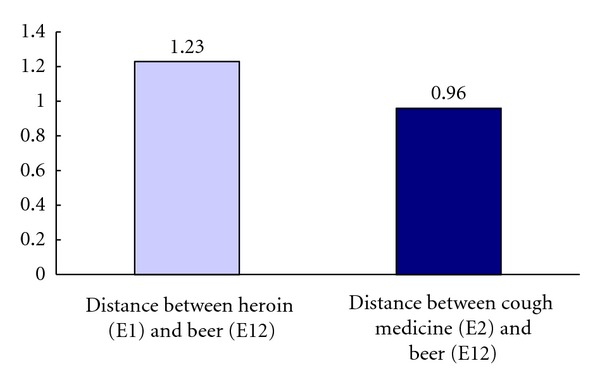
Mean distance between heroin (E1) and beer (E12) versus mean distance between cough medicine (E2) and beer (E12).

**Figure 4:**
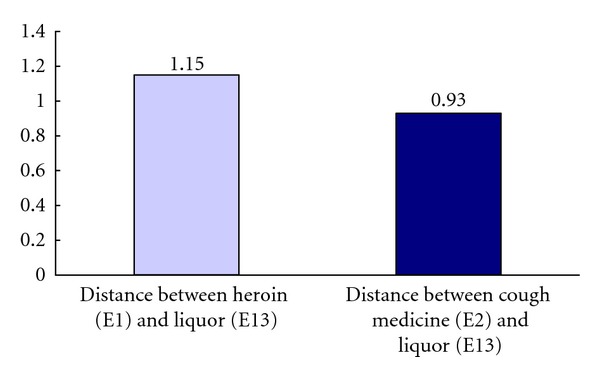
Mean distance between heroin (E1) and liquor (E13) versus mean distance between cough medicine (E2) and liquor (E13).

- Analyses using Wilcoxon Test showed that the mean distance between element 1 (heroin) and element 11 (cigarette) was significantly longer than the mean distance between element 2 (cough medicine) and element 11 (cigarette): *z* = −2.22, *P* < .05. In other words, relative to the construed similarity between heroin and cigarette, the informants construed cough medicine to be more similar to cigarette. The difference is graphically presented in Figure 2.
- Analyses using Wilcoxon Test showed that the mean distance between element 1 (Heroin) and element 12 (Beer) was significantly longer than the mean distance between element 2 (cough medicine) and element 11 (Beer): *z* = −2.31, *P* < .05. In other words, relative to the construed similarity between heroin and beer, the informants construed cough medicine to be more similar to beer. The difference is graphically presented in Figure 3.
- Analyses using Wilcoxon Test showed that the mean distance between element 1 (heroin) and element 13 (liquor) was significantly longer than the mean distance between element 2 (cough medicine) and element 13 (liquor): *z* = −2.05, *P* < .05. In other words, relative to the construed similarity between heroin and liquor, the informants construed cough medicine to be more similar to liquor. The difference is graphically presented in Figure 4.

**Figure 5:**
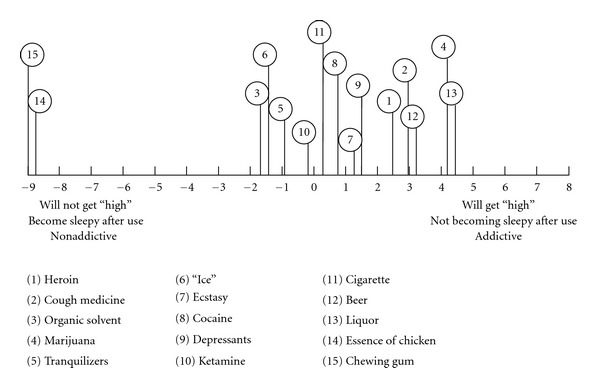
Representation of cough medicine and other drugs and non-drugs in the psychological space in terms of the first principal component (informant no. 3).

An illustration based on case no. 3 can give some ideas on the informant's construction of cough medicine in relation to the non-drugs. The informant aged 32, and he had tried heroin, marijuana, tranquilizers, “ice”, and cough medicine. He began abusing cough medicine at the age of 22 with a usual consumption of 2–6 times every week. He had abused that cough medicine 2–6 times each week in the past three months. With reference to Table 4, the informant construed cough medicine had the following characteristics after consumption: creating weak sexual impulse, feeling “high,” feeling down, bringing quick psychological reaction, addictive, feeling good, feeling relaxed, easy to sleep, relatively expensive, and having greater harm on the body. The informant's representation of cough medicine and other drugs and non-drugs in his psychological space is presented in Figure 5. In the figure, it is evident that the informant construed cough medicine to be similar to beer (element 12) and liquor (element 13).

**Figure 6:**
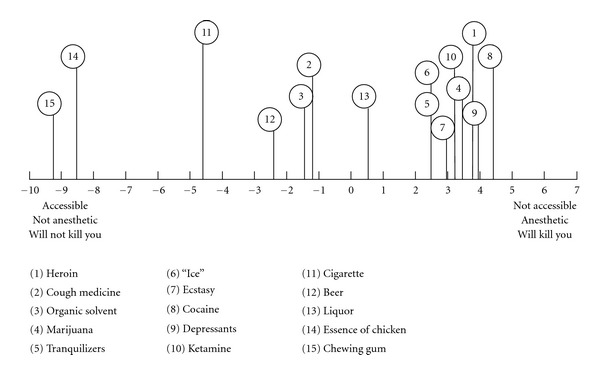
Representation of cough medicine and other drugs and non-drugs in the psychological space in terms of the first principal component (informant no. 10).

Another illustration based on case no. 10 can also give some ideas on the informant's construction of cough medicine in relation to the non-drugs. The informant aged 34, and he had only abused cough medicine. He began abusing cough medicine at the age of 31. His usual consumption of cough medicine was 2–6 times each week, and he had abused cough medicine once a week in the past three months. With reference to Table 5, the informant perceived that cough medicine had the following characteristics: easily addicted, expensive, feeling “high” after consumption (2 times), easy to get, not feeling faint after consumption, losing self-control if taken excessively, not feeling dull after consumption, feeling sedated after consumption, and feeling excited after consumption. The informant's representation of cough medicine and other drugs and non-drugs in his psychological space is presented in Figure 6. In the figure, it is evident that while the informant construed cough medicine to be similar to beer (element 12) and liquor (element 13), cough medicine was seen to be different from most of the drugs (except organic solvents). 

Pearson correlation analyses showed that a higher level of construed similarity between cough medicine and the gateway drugs was related to a lower level of construed harm of cough medicine (*r* = .33, *P* = .33 for cigarette, *r* = .70, *P* < .05 for beer, and *r* = .53, *P* < .10 for liquor). In other words, for those who construed that there was high dissimilarity between cough medicine and the gateway drugs, they tended to perceive that cough medicine had greater harm.

## 4. Discussion

With a few exceptions, there are few studies on cough medicine abuse in the Chinese contexts. Shek and Lam [48] examined beliefs about cough medicine abuse among Chinese young people using the Beliefs about Cough Medicine Abuse Scale (BACMAS), with a total of 225 Chinese young people (160 cough medicine abusers and 65 noncough medicine abusers) participated in the study. Results showed that the scale was internally consistent. The scale was able to differentiate those who abused and did not abuse cough medicine, and a higher level of BACMAS scores was related to higher levels of endorsement of cough medicine abuse and severity of consumption, thus providing support for the concurrent and construct validities of the scale. Regarding attitudes to cough medicine abuse, although the respondents abusing cough medicine generally did not perceive any benefits of abusing cough medicine and they recognized the harmful effects of cough medicine abuse, roughly three-tenth of the respondents believed that cough medicine was not addictive, and more than half of them believed that there was no harm to get along with friends abusing cough medicine.

Besides, qualitative data on the views of different stakeholders on cough medicine abuse have been reported. Lam and Shek [49] conducted focus groups based on young cough medicine abusers (*N* = 8), parents of cough medicine abusers (*N* = 5), and social workers (*N* = 6). Several observations were revealed from the qualitative findings. First, all the research participants shared a view that their first use of cough medicine was under the influence of peers, and they remarked that they had no problems at all to obtain legally controlled cough medicine from pharmacies. Although there were no obvious discomforts at the initial stage, the participants reported observed harmful effects in the physical, psychological, interpersonal, and family domains after prolonged cough medicine use. Second, social factors (mainly peer influence), personal factors (avoidance), family factors (poor family relationship or unhappy family incidents), availability (ease of access), and ignorance (unawareness of the harmful consequences and wrong beliefs) were identified to be the reasons for cough medicine abuse. In particular, family members were generally insensitive to the issue of cough medicine abuse. Emotional and financial problems were major difficulties encountered by the families concerned. Third, there were personal (dependency and wrong beliefs), environmental (accessibility of cough medicine and ignorance of service), and system barriers (loose legal control and stigmatization) to help seeking. Personal factors (self-awareness and insight) and support systems (family and service support) were identified to be the factors facilitating help seeking. Finally, the stakeholders and service providers recommended that there was the need for holistic treatment orientation, collaboration among helping professions, and reaching out. It was also recommended that the government should be more proactive in community education and stricter law enforcement, and separate hospital arrangements should be made to avoid stigmatization. Although the focus group findings are very revealing, the findings are more related to social construction of cough medicine abuse in the focus groups instead of personal construction of cough medicine abuse.

Against this background, the present study was conducted to understand personal constructions of cough medicine, and a personal construct theory was adopted to examine the issue concerned. There are several conclusions of the study. First, personal constructions of cough medicine were mixed, and the observation is in line with the findings reported by Shek and Lam [48] and Lam and Shek [49]. Practically, it is important to further understand why abusers continue to abuse cough medicine despite their realization of its harmful effect. In addition, it is also important to consider how the construed benefits of cough medicine abuse can be minimized, whereas the construed harms can be translated into motivation to quit the drug. Second, regarding the addictive nature of cough medicine, although the informants perceived cough medicine to be additive and harmful, they perceived cough medicine to be less addictive and less harmful than heroin. The finding suggests that the distinction between “hard” and “soft” drugs is still in the minds of the abusers. Third, concerning cough medicine versus other drugs, while the informants construed cough medicine to be similar to ketamine and marijuana, they also perceived cough medicine to possess some characteristics of heroin. Because cough medicine abusers tend to construe cough medicine as similar to soft drugs, more preventive education and publicity work should be done. While there are anti-heroin and antipsychotropic substances (such as ecstasy and ketamine) initiatives, there are comparatively fewer anti-cough medicine abuse preventive and publicity attempts, such as APIs and posters.

Fourth, regarding cough medicine and drugs versus other drugs, relative to the construed similarity between heroin and the gateway drugs (cigarette, beer, and liquor), the informants construed cough medicine to be more similar to the gateway drugs. Again, the theme of relatively “milder” harm of cough medicine abuse in comparison with heroin can be seen from the findings. The finding suggests that the abusers might be psychologically less conscious of the unique addictive and harmful effects of cough medicine. More efforts to reduce the “blurred” nature of cough medicine are needed. Fifth, for those who construed that there was high dissimilarity between cough medicine and the gateway drugs, they tended to perceive that cough medicine had greater harm. The finding suggests that there is a need to change the personal constructions of cough medicine abusers. Sixth, consistent with the spirit of personal construct psychology, the present findings suggest that there is a need for helping professionals to thoroughly understand the beliefs and personal construct systems of the abusers of cough medicine. Therapeutically speaking, how to change the personal construction and beliefs about cough medicine of the abusers is an important area for intervention. Finally, the present findings underscore the usefulness of using the repertory grid technique in understanding the subjective worlds of the abusers regarding different types of drugs, including their perceived benefits and harms.

In conjunction to the findings of the present study, several recommendations regarding prevention of cough medicine abuse are proposed. First, as the cough medicine abusers are not fully aware of the addictive nature of cough medicine, there is a need to raise the public awareness of the harmful effects of cough medicine via preventive education and publicity initiatives as follows: (a) public, school-based, and family-based preventive education should be carried out, particularly in families with young adolescents; (b) young people should be helped to develop nonpermissive and proper beliefs about cough medicine abuse; (c) in view of the relative lack of preventive education and publicity materials on anti-cough medicine abuse (e.g., posters and APIs), the government should review the existing anti-cough medicine initiatives and develop timely measures.

Besides, young substance abusers should understand the negative impact of cough medicine abuse. Some researchers have studied the perceived effects of abusing cough medicine. Based on a group of Indian abusers, Mattoo et al. [18] found the following reported effects of taking cough medicine: feeling alert, becoming more active, feeling cheerful, becoming overtalkative, and feeling drowsy. Based on the data collected via focus group discussion, Sung [50] found that cough medicine abusers perceived the following benefits of taking cough medicine: enhancement of concentration on routine jobs, ability to relax, and enhancement of energy and excitement. There are other studies showing that abuse of cough medicine produces psychotic states. Krenzelok [51] reported that intake of a large amount of cough syrup might produce phencyclidine- (PCP-) like effect, hyperactivity, and hallucination. Mendez [52] suggested that sympathomimetic amines in cough medicine could lead to manic states. With specific reference to dextromethorphan, which is a common ingredient in cough medicine, Fisher [53] commented that if taken in large quantity, dextromethorphan would produce PCP-like effect. Price and Liebel [54] reported a case of dextromethorphan-induced psychosis and concluded that “clinicians should be vigilant in treating cases that suggest dextromethorphan abuse” (p.304). Polles and Griffith [55] reported two cases showing that dextromethorphan could cause secondary mania. Bostwick [56] also summarized “the take-home message is that OTC preparations are a potential psychoactive polypharmacy in a bottle and that should be used with caution in patients with predisposition to affective illness by family or personal history” (p.572). In a recent study of cough medicine abuse in Hong Kong, Au et al. [57] examined four cases of young men with cough mixture abuse and severe folate deficiency. The four cases presented at least two of following symptoms: difficulty in walking, dizziness, lower limb weakness, numbness in all limbs, axonal degeneration, and dental caries, with the last two symptoms found in most cases.

In addition to preventive drug education and understanding of the nature of cough medicine abuse, promotion of psychosocial competence is also important. As the present findings showed that cough medicine abusers held contradictory beliefs about cough medicine, strengthening of their cognitive competence is indispensable. In particular, young people should be helped to understand the role of peers in cough medicine abuse (i.e., social competence). Resistance skills (i.e., behavioral competence) and healthy network building (i.e., bonding) should be emphasized. Besides, strategies that can enhance the coping abilities (i.e., resilience) and reduce the curiosity of young people about drugs should be devised. Young people should be helped to understand the role of family factors in cough medicine abuse, and they should learn how to deal with negative emotions about their families (i.e., emotional competencies). In the west, many programs have been developed to promote the psychosocial competencies of young people to stay away from drugs. Nevertheless, while there are successful programs to reduce substance abuse in young people in the west, related effective programs are almost nonexistent in the Chinese contexts [58]. One notable exception is the project P.A.T.H.S. in Hong Kong. There are evaluation findings showing that the program was able to promote positive youth development and reduce substance abuse and delinquency of participants [59–63]. It is suggested that there is a need to further promote the program and increase the sustainability of the program in the long run.

There are several contributions of the present research project. First, because no systematic study of cough medicine abuse has been carried out in different Chinese and in global contexts, this is a groundbreaking attempt, and the present findings contribute to the limited literature. Second, the present study provides additional evidence suggesting that substance abuse among young people is a multidimensional problem involving different psychosocial factors. Third, the present study underscores the importance of triangulation in the understanding of cough medicine abuse. Fourth, the present study demonstrates the importance of understanding the psychological worlds of cough medicine abusers. Finally, the scale-assessing beliefs about cough medicine abuse are invaluable to drug prevention workers in different Chinese contexts.

Nevertheless, there are also several limitations of the present study. First, because of the cross-sectional design of this research, we need to further investigate the causal processes involved in cough medicine abuse among young people via longitudinal studies. Second, a purposive sample design was adopted in this study. While this design is commonly used in research on drug abuse, its limitations, particularly with respect to the generalizability of the findings, should be duly acknowledged. Third, the sample size of the present study is relatively small, as only 11 informants were recruited, which will also affect the generalization of findings. However, this sample size is considered reasonable in qualitative study. Fourth, if resources permit, participants randomly drawn should be recruited to participate in the repertory grid tests. In particular, a wider range of cough medicine abusers and their parents as well as social workers should be recruited to participate in the related studies. Despite these limitations, this study has provided the most updated information to enrich our understanding of cough medicine abuse among young people in Hong Kong. The policy and service implications of the present findings deserve the attention of young people, parents, teachers, helping professionals, policy makers, and the general public. 

## Figures and Tables

**Table 1:** Personal constructions of cough medicine among the informants.

Informant	Personal construction
(1)	Easy to get addicted, not easily get fainted after consumption, confused after consumption, drug effect not so strong, not good, will make me addicted, relatively cheap, and difficult to get
(2)	Not good for body, not good thing, harmful to body, expensive, accessible, addictive, has a bad influence to body, and illegal to consume
(3)	Sexual urge created by the drug and is not strong, getting “high” after consumption, sleepy after consumption, drug effect strong, addictive, good feeling after consumption, making oneself relaxed, easy to fall asleep after consumption, relatively expensive, and relatively harmful to body
(4)	Makes one become excited, spirit lifting, can control oneself after consumption, makes one feel relaxed, no need to think much after consumption, feeling less stress after consumption, will not feel unhappy after consumption, and addictive
(5)	Not feeling painful after consumption, not feeling faint after consumption, becomes calm after consumption, feeling comfortable after consumption, feeling confused after consumption, feeling relaxed after consumption, want to move after consumption, knowing what one is doing after consumption, harmful to body, and feeling at home after consumption
(6)	Good feeling after consumption, will paralyze you, unconscious after consumption, getting high, will become more unrestricted after consumption, will not lose control, will become braver, more concentrated in work, and mind will not become unclear
(7)	Comfortable after consumption, will become confused, will become high, enjoyable, will become excited, relatively toxic, not painful after consumption, and harmful
(8)	Addictive, getting high after consumption, spirit lifting, not feeling irritable, cheap, good spirit after consumption, confused mind after consumption, poor memory after consumption, and feeling happy after consumption
(9)	Paralyzed after consumption, concentrated after consumption, feeling good, will make closed ones feel sad, bad for body, forget about worries after consumption, like it, easy to quit, and completely conscious
(10)	Addictive, using more money, will get high after taking it, accessible, easily get fainted, will not lose control, will not become dull, anesthetic, and will become excited after consumption
(11)	Addictive, will not get confused after consumption, remains clear-minded after consumption, feeling good after consumption, many young people take it, not hallucinated after consumption, like it, comfortable after consumption, and harm is great

**Table 2:** Mean distances between cough medicine and other drugs.

Pair	Mean distance
Cough medicine and heroin	.72
Cough medicine and organic solvent	.84
Cough medicine and marijuana	.68
Cough medicine and tranquilizers	.76
Cough medicine and “ice”	.71
Cough medicine and ecstasy	.73
Cough medicine and cocaine	.75
Cough medicine and depressants	.81
Cough medicine and ketamine	.67

**Table 3:** The completed grid form of informant no. 7.

Elements	(1) Heroin	(2) Cough medicine	(3) Organic solvent	(4) Marijuana	(5) Tranquilizers	(6) “Ice”	(7) Ecstasy	(8) Cocaine	(9) Depressants	(10) Ketamine	(11) Cigarettes	(12) Beer	(13) Liquor	(14) Essence of chicken	(15) Chewing gum	Construct pole	Contrast pole
(a)	5	6	3	4	5	5	4	6	5	5	4	4	2	6	4	Comfortable	Uncomfortable
(b)	3	6	3	4	4	6	6	5	4	5	1	4	4	1	1	Confused	Not confused
(c)	5	6	5	4	5	6	6	6	5	5	2	4	6	1	1	Become high	Not becoming high
(d)	5	6	4	5	5	6	6	6	5	6	3	4	3	1	1	Enjoyable	Not enjoyable
(e)	5	6	4	5	5	6	5	6	5	6	3	4	4	1	3	Excited after use	Not excited after use
(f)	6	5	6	3	5	6	5	5	5	5	2	4	5	1	1	Toxic	Less toxic
(g)	1	1	4	2	1	2	1	1	1	1	1	5	5	1	1	Painful after use	Not painful after use
(h)	5	6	3	4	5	5	4	6	5	5	4	4	2	6	4	Comfortable	Uncomfortable
(i)	1	1	4	2	1	2	1	1	1	1	1	5	5	1	1	Painful after use	Not painful after use
(j)	4	5	1	4	4	1	2	2	2	2	6	5	5	6	6	Harmful	Less harmful
(k)	6	3	3	3	2	3	2	4	5	5	1	2	2	1	1	Addictive	Not addictive
(l)	6	6	5	4	4	6	6	5	5	5	1	2	3	1	1	Will kill you	Will not kill you

**Table 4:** The completed grid form of informant no. 3.

Element	(1) Heroin	(2) Cough medicine	(3) Organic solvent	(4) Marijuana	(5) Tranquilizers	(6) “Ice”	(7) Ecstasy	(8) Cocaine	(9) Depressants	(10) Ketamine	(11) Cigarettes	(12) Beer	(13) Liquor	(14) Essence of chicken	(15) Chewing gum	Construct pole	Contrast pole
(a)	5	3	1	4	2	6	1	6	2	2	1	4	4	6	1	Strong sexual urge	No strong sexual urge
(b)	5	4	1	6	1	1	6	5	4	4	3	5	5	1	1	Getting high	Not getting high
(c)	3	5	1	5	4	1	2	3	6	3	3	5	6	1	1	Sleepy	Not Sleepy
(d)	5	5	6	5	1	4	3	4	5	4	4	5	5	1	1	Strong drug effect	Weak drug effect
(e)	6	5	5	6	6	4	6	4	6	4	6	6	4	1	1	Addictive	Nonaddictive
(f)	4	4	3	6	4	4	4	4	5	4	5	6	4	1	1	Feeling good	Not feeling good
(g)	3	6	1	3	3	1	1	3	1	4	4	6	5	1	1	Relaxed	Not relaxed
(h)	1	4	1	4	5	1	1	3	1	1	1	5	6	1	1	Easy to sleep	Not easy to sleep
(i)	1	3	4	5	6	1	1	1	3	2	5	4	1	1	6	Cheap	Expensive
(j)	6	5	6	6	4	6	6	4	4	4	5	3	6	1	1	Harmful to body	Less harmful to body
(k)	6	6	4	4	5	5	6	5	6	5	6	5	5	1	2	Addictive	Not addictive
(l)	6	4	5	6	4	6	6	5	4	4	3	3	6	1	1	Will kill you	Will not kill you

**Table 5:** The completed grid form of informant no. 10.

Elements	(1) Heroin	(2) Cough medicine	(3) Organic solvent	(4) Marijuana	(5) Tranquilizers	(6) “Ice”	(7) Ecstasy	(8) Cocaine	(9) Depressants	(10) Ketamine	(11) Cigarettes	(12) Beer	(13) Liquor	(14) Essence of chicken	(15) Chewing gum	Construct pole	Contrast pole
(a)	6	4	3	3	5	5	2	6	3	4	2	2	4	1	1	Addictive	Not addictive
(b)	5	4	2	6	3	5	4	6	4	4	3	2	5	4	1	Using more money	Not using much money
(c)	3	4	4	5	5	4	6	5	6	5	2	4	5	1	1	Getting “high”	Not getting “high”
(d)	6	1	1	6	4	6	4	6	4	4	1	1	1	1	1	Not accessible	Accessible
(e)	3	4	4	5	5	4	6	5	6	5	2	4	5	1	1	Getting “high”	Not getting “high”
(f)	4	3	4	6	5	1	5	4	5	6	3	3	4	1	1	Easy to faint	Not easy to faint
(g)	6	4	4	5	4	5	5	4	5	5	3	4	5	1	1	Will lose control	Will not lose control
(h)	4	3	4	5	5	3	6	4	6	6	3	4	4	1	1	Become dull	Not becoming dull
(i)	5	4	4	5	5	4	6	5	6	4	2	4	5	1	1	Anesthetic	Nonanesthetic
(j)	4	3	5	4	6	2	4	4	4	4	4	4	5	6	6	Not excited	Excited
(k)	6	4	3	3	5	5	2	6	3	4	2	2	4	1	1	Addictive	Not addictive
(l)	6	3	4	4	4	6	5	5	6	5	3	3	3	1	1	Will kill you	Will not kill you

## References

[1] Jensen S, Hansen AC (1993). Abuse of codeine separated from over-the-counter drugs containing acetylsalicylic acid and codeine. *International Journal of Legal Medicine*.

[2] Jinks KJ, Raschko RR (1990). A profile of alcohol and prescription drug abuse in a high-risk community-based elderly population. *Drug Information in Clinical Practice*.

[3] Students for Sensible Drug Policy Cold Medicine Abuse Spreading Among Youths: Officials Finds. http://www.mapinc.org.

[4] National Institute on Drug Abuse (2011). *High School and Youth Trends*.

[5] http://www.teenoverthecounterdrugabuse.com.

[6] Anonymous (1985). Cough medicines. *Drug Therapy Bulletin*.

[7] BBC Online Network Health Pharmacist’s Painkiller Warning. http://www.bbc.co.uk/news/.

[8] Darboe MN (1996). Abuse of dextromethorphan-based cough syrup as a substitute for licit and illicit drugs: a theoretical framework. *Adolescence*.

[9] Darboe MN, Keenan GR, Richards TK (1996). The abuse of dextromethorphan-based cough syrup: a pilot study of the community of Waynesboro, Pennsylvania. *Adolescence*.

[10] Office on Drugs and Crime, United Nations Fact Sheets: Drug Abuse Trends in East Asia. http://www.unodc.org.

[11] Office on Drugs and Crime, United Nations Regional Centre for East Asia and the Pacific. Fact Sheets: Opium and Derivatives. http://www.unodc.org.

[12] Bureau for International Narcotics and Law Enforcement Affairs International Narcotics Control Strategy Report: 2002. http://www.state.gov.

[13] Medical Tribune Online Codeine Cough Mixtures Banned from Next Year. http://www.medicaltribune.com.

[14] Ishigooka J, Yoshida Y, Murasaki M (1991). Abuse of “BRO NO”: a Japanese O.T.C. cough suppressant solution containing methylephedrine, codeine, caffeine and chlorpheniramine. *Progress in Neuro-Psychopharmacology and Biological Psychiatry*.

[15] Suzuki T, Masukawa Y, Misawa M (1990). Drug interactions in the reinforcing effects of over-the-counter cough syrups. *Psychopharmacology*.

[16] Borde M, Nizamie SH (1988). Dependence on a common cough syrup. *Lancet*.

[17] Wairagkar NS, Das J, Kumar S (1994). Codeine containing cough syrup addiction in Assam and Nagaland. *Indian Journal of Psychiatry*.

[18] Mattoo SK, Basu D, Sharma A, Balaji M, Malhotra A (1997). Abuse of codeine-containing cough syrups: a report from India. *Addiction*.

[19] Narcotics Division (2010). *Central Registry of Drug Abuse (Fifty-eight report)*.

[20] Lau JTF (2002). *The 2000 Survey of Drug Use among Students*.

[21] Narcotics Division (1991). *1990 Survey on the Non-Medical Use of Psychotropic Substances among Students of Secondary Schools and Technical Institutes*.

[22] Narcotics Division (1993). *1992 Survey on Drug Use among Students of Secondary Schools and Technical Institutes*.

[23] Narcotics Division (1997). *1996 Survey on Drug Use among Students of Secondary Schools and Technical Institutes*.

[24] Narcotics Division (2010). *Central Registry of Drug Abuse (Fifty-Ninth Report)*.

[25] Narcotics Division (2010). *The 2008/09 Survey of Drug Use among Students*.

[26] Narcotics Division (1994). *Report on Survey of Young Drug Abusers*.

[27] Narcotics Division (1994). *1994 Survey to Assess Public Awareness of Anti-Drug Activities*.

[28] Lai B (1997). *A Retrospective and a Prospective Study of Psychoactive Substance Abusers of PS33*.

[29] Day J, Lee C (1996). *An Evaluation of Talks Given by the Narcotics Division, Hong Kong Government Secretariat, to Students in Secondary Schools, for Prevention of Drug Abuse*.

[30] Lam LCW, Lee D, Shum PPS, Chen CN (1996). Cough mixture misuse in Hong Kong—an emerging psychiatric problem?. *Addiction*.

[31] Wong CSY, Tang CSK, Schwarzer R (1997). Psychosocial correlates of substance use: comparing high school students with incarcerated offenders in Hong Kong. *Journal of Drug Education*.

[32] Caritas Aberdeen Outreaching Team (2000). *Substance Abuse among Young People in Discos*.

[33] Narcotics Division (2002). *Report of the Task Force on Psychotropic Substances Abuse*.

[34] Chiu S, Wong V (2002). *A Research Study on Substance Abuse among Young People in Yuen Long*.

[35] Kelly G (1955). *The Psychology of Personal Constructs (Vols. 1-2)*.

[36] Fransella F, Fransella F (1981). Repertory grid technique. *Personality: Theory, Measurement and Research*.

[37] Fransella F, Bannister D (1977). *A Manual for Repertory Grid Technique*.

[38] Viney LL (1998). Should we use personal construct therapy? A paradigm for outcomes evaluation. *Psychotherapy*.

[39] Shek DTL, Lam CW Evaluation of a preventive drug education program in Hong Kong using the repertory grid test.

[40] Slater P (1976). *The Measurement of Interpersonal Space by Grid Technique*.

[41] Slater P (1977). *The Measurement of Interpersonal Space by Grid Technique*.

[42] Bannister D (1965). The rationale and clinical relevance of repertory grid technique. *The British Journal of Psychiatry*.

[43] Shaw MLG (1981). *Recent Advances in Personal Construct Technology*.

[44] Norris H, Makhlouf-Norris F, Slater P (1976). The measurement of self-identity. *Explorations of Intrapersonal Space*.

[45] Stanley B, Beail N (1985). Alienation in young offenders. *Repertory Grid Technique and Personal Constructs: Applications in Clinical and Educational Settings*.

[46] Luk AL, Shek DTL (2006). Perceived personal changes in Chinese ex-mental patients attending a holistic psychiatric rehabilitation program. *Social Behavior and Personality*.

[47] Shek DTL Evaluation of a positive youth development program based on the repertory grid test.

[48] Shek DTL, Lam CM (2008). Beliefs about cough medicine abuse among Chinese young people in Hong Kong. *Social Behavior and Personality*.

[49] Lam CM, Shek DTL (2006). A qualitative study of cough medicine abuse among Chinese young people in Hong Kong. *Journal of Substance Use*.

[50] Sung E (2001). *A focus group study on psychotropic substance abuse*.

[51] Krenzelok EP (1990). Non-prescription cough medicine abuse. *Clinical Toxicology Forum*.

[52] Mendez MF (1992). Mania self-induced with cough syrup. *Journal of Clinical Psychiatry*.

[53] Fisher JD (1991). Dextromethorphan. *Clinical Toxicology Review*.

[54] Price LH, Liebel J (2000). Dextromethorphan-induced psychosis. *American Journal of Psychiatry*.

[55] Polles A, Griffith JL (1996). Dextromethorphan-induced mania. *Psychosomatics*.

[56] Bostwick JM (1996). Dextromethorphan-induced manic symptoms in a bipolar patient on lithium. *Psychosomatics*.

[57] Au WY, Tsang J, Cheng TS (2003). Cough mixture abuse as a novel cause of megaloblastic anaemia and peripheral neuropathy. *British Journal of Haematology*.

[58] Shek DTL, Yu L (2011). A review of validated youth prevention and positive youth development programs in Asia. *International Journal of Adolescent Medicine and Health*.

[59] Shek DTL, Sun RCF (2010). Subjective outcome evaluation based on secondary data analyses: the project P.A.T.H.S. in Hong Kong. *The Scientific World Journal*.

[60] Shek DTL, Ng CSM, Tsui PF (2010). Qualitative evaluation of the project P.A.T.H.S.: findings based on focus groups. *International Journal on Disability and Human Development*.

[61] Shek DTL (2010). Using students’ weekly diaries to evaluate positive youth development programs: are findings based on multiple studies consistent?. *Social Indicators Research*.

[62] Shek DTL (2010). Quantitative evaluation of the training program of the project P.A.T.H.S. in Hong Kong. *International Journal of Adolescent Medicine and Health*.

[63] Shek DTL, Sun RCF (2010). Secondary data analyses of subjective outcome evaluation findings of the project P.A.T.H.S. in Hong Kong. *The Scientific World Journal*.

